# Evaluating Mobile Apps Targeting Older Adults: Descriptive Study

**DOI:** 10.2196/37329

**Published:** 2023-04-27

**Authors:** Megan Sweeney, William Barton, Camille Nebeker

**Affiliations:** 1 Herbert Wertheim School of Public Health and Human Longevity Science University of California, San Diego La Jolla, CA United States; 2 Research Center for Optimal Digital Ethics in Health University of California, San Diego La Jolla, CA United States

**Keywords:** older adults, mobile apps, privacy, data management, research ethics, app, aging, environment, safety, smartphone, personal information, user knowledge, user, data, data collection, storage

## Abstract

**Background:**

Smartphone use has increased dramatically and, in parallel, a market for mobile apps, including health apps, has emerged. The business model of targeted mobile app advertisements allows for the collection of personal and potentially sensitive information, often without user knowledge. Older adults comprise a rapidly growing demographic that is potentially vulnerable to exploitation by those accessing data collected via these apps.

**Objective:**

This research examined apps that claimed to be useful to older adults with a goal of (1) classifying the functionality of each app, (2) identifying whether a privacy policy existed and was accessible, and (3) evaluating evidence that could support claims of value to older adults.

**Methods:**

An environmental scan was conducted using the Google search engine and typing “apps for older adults.” The first 25 sites that this search returned comprised the primary data for this study. Data were organized by descriptive features of purpose (eg, health, finance, and utility), the existence of an electronically accessible privacy policy, price, and evidence supporting each recommended mobile app.

**Results:**

A total of 133 mobile apps were identified and promoted as being the best “apps for older adults.” Of these 133 mobile apps, 83% (n=110) included a privacy policy. Fewer apps designated in the “medical” category included a privacy policy than those classified otherwise.

**Conclusions:**

The results suggest that most mobile apps targeting older adults include a privacy policy. Research is needed to determine whether these privacy policies are readable, succinct, and incorporate accessible data use and sharing practices to mitigate potential risks, particularly when collecting potentially sensitive health information.

## Introduction

The smartphone has created a market for a myriad of mobile apps, including health apps, that play a central in role our daily lives. At present, both Android and Apple offer around 2 million apps for public download [1]. Many of these apps require the collection of user personal data to access services. This information is used to support service delivery, including tailoring algorithms, which produce targeted advertisements customized to app users. It is unlikely that most app users are fully aware of the nature and scope of personal data collected or how those data are used in targeted advertisements and monetization of apps [2]. By activating an app, users agree to disclose personal information including, for example, demographics, contacts, health, and lifestyle details to access the app services [2]. The collection of personal and potentially sensitive information may pose unforeseen risks to the app users. Older adults are especially vulnerable to exploitation due to low technology and data literacy. Moreover, prior research has demonstrated that most older adults are unfamiliar with the extent of personal information that they agree to share when using various apps [3,4].

The issue of data sharing is intensified for older adults, a rapidly increasing demographic that has previously demonstrated technology literacy levels far lower than that of younger generations [5,6]. In a 2019 paper, Wang et al [7] discussed how a cohort of seniors living in a retirement community felt uncomfortable working with technology and primarily relied on grandchildren for technological support. Nonetheless, leveraging a younger or more “tech-savvy” family member is not always an accessible resource for the 65 years and older demographic. When asked what future technologies this group of older adults thought would be helpful, some participants suggested features they already had access to but may not have been aware of how to use (eg, universal remote) [7]. This lack of familiarity with technology can put seniors and other vulnerable populations at risk.

Currently, there is a relatively low threshold for app developers to meet when putting an app into the Apple App Store or Google Play Store [8]. Apps that become available to the public on the Apple App Store or Google Play Store often lack basic security or data protection measures—with prior reports citing as many as 49% of apps fail basic data protection capabilities [9]. With the vast quantity of apps available for download combined with the lack of safety precautions, it is possible for personal information to be misused and compromise individuals’ privacy by sharing individual data or installing malware through mobile apps [10,11]. This is concerning for older adults, who have varying levels of digital literacy and may be unaware of the data sharing and privacy risks associated with mobile applications [5]. A study of the 1100 most popular Android apps revealed misuse of personal information [8]. It found that phone identifiers, which are distinct digit combinations used to distinguish individual devices, could track web-based activity back to an individual person, and in some cases, without permission of the app users. The study also found that advertising and analytic companies collect, buy, and sell data due to insufficient protection of sensitive information by the apps that initially collect these data [8]. While the researchers found no evidence of harmful malware, the large misuse of information poses potential risks of harm.

Several factors influence behaviors and attitudes surrounding data sharing, including privacy preferences [7]. In general, privacy, transparency, and full disclosure of information sharing are highly important to older adults [12]. Many seniors, nonetheless, are willing to share their information to delegate control to others or to gain something in return for sharing their data, as long as they know the information that is being disclosed and retain granular control [13]. This perspective aligns with the business models of apps that offer a service in exchange for the information that they collect. On the other hand, it is unclear whether the user understands the nature and granularity of information being collected when initiating app use, as well as information collected longitudinally while using the app.

Privacy policies generally disclose what information is collected prior to downloading the application and how those data will be used and shared. That being said, privacy policies are lengthy and unreadable for the average adult [14,15]. The average adult in the United States tends to read at an eighth-grade reading level [16,17]. A study by Das et al [18], however, found the average reading grade level of apps targeting youth to be 12.78, which is equivalent to a first-year college student. This gap between the reading level of adults and privacy policy language complexity poses a barrier to understanding what personal information is collected and shared. Moreover, not all apps include a privacy policy, in which case, the app user does not have access to the data collection and sharing practices [19,20].

As the COVID-19 pandemic led to stay-at-home orders and social distancing, older adults found themselves more isolated. During this time, social technologies became increasingly important in supporting healthy aging. The use of these technologies by older adults requires an awareness of associated benefits as well as possible harms, including risks related to terms of service and privacy protocols [21]. Social isolation and loneliness are important to prevent as they can lead to higher risks of cognitive impairment and can even result in the onset of vascular and neurological diseases [22,23]. A potential solution to mitigate harms related to pandemic quarantine isolation includes access to technology. Daly et al [24] found that technologies are key in preserving social connectivity among older adults, but they also acknowledged that older adults’ lack of technological literacy presented a barrier to technology effectiveness.

The simple interface of mobile apps could provide an accessible option for older adults to stay connected; yet, little is known about mobile apps that are specifically targeting older adults. This research examined apps that claimed to be useful to older adults with a goal of (1) classifying the functionality of each app, (2) identifying whether a privacy policy existed and was accessible, and (3) evaluating evidence that could support claims of value to older adults.

## Methods

To identify mobile apps targeting older adults, a search was conducted using the terms “apps for older adults” via the Google search engine. Given that only 2%-3% of people who conduct Google searches look past the first page, we limited our data collection to the first 25 links, which was about the first 2.5 pages of Google search results [25]. Each link included upwards of 10 app recommendations, with some listing around 40, which provided a sufficient corpus of data to analyze.

We found that the links produced by a Google search change frequently, especially after the first page. To counter this, screenshots of the first 3 pages, captured on July 19, 2019, were saved and used as our primary data source. The first 5 results given on page 1 of our Google search were used to identify key descriptive variables to record in our classification system including price, category (eg, *Medical*, *Entertainment*), the existence of a privacy policy, evidence supporting the app recommendation, App Store rank within each predefined category, and availability on Apple or Android devices. Our preliminary search demonstrated that predefined categories and rankings by Apple and Android differed considerably. This difference prompted us to limit our data collection to apps available on the Apple App Store and developed for use on an iPhone. Thus, we excluded 8 of the recommended apps—including 5 that were exclusive to the Google Play Store, as well as 3 apps that were developed for only Mac or PC and unavailable on a mobile device.

Each of the first 25 links was reviewed, and descriptive data were entered into an Excel (Microsoft Corp) spreadsheet that included both classifications for recommended apps on the app store, free or paid status, ranking within its category, evidence of a vetting process, and the existence of a privacy policy. For an app to be classified as “vetted” per our standards, a website recommending the app must have provided some explanation as to why these apps were chosen that was backed by evidence. Whether the app was supported by evidence was determined by reading the websites and noting if there was any explanation about how the app was selected for use by older adults. There were several occasions where an app was listed on more than one of the websites produced by our search. Across all of the sites with app recommendations in our analysis, 17 apps were recommended by 3 distinct sites, and 28 apps were recommended by 2 distinct sites. In these cases, the duplicate apps were only recorded once.

It is important to note that some of the apps recommended by websites had changed their official titles at the time of the search from the time they were recommended. Others no longer existed in the app store. Those unable to be located were not included in the data set. Whether the application required payment was not always clear, as only a few of the apps required money at the first step to download from the Apple App Store. However, there were many instances when an in-app purchase was necessary for the app to be fully used. According to the Apple App Store, keywords, such as “subscription,” denoted a subscription-based fee required to fully use the app, while words such as “pro” or “premium” implied that there was a free, usable, but limited version (often labeled the “lite” version), with the option for a paid ”pro” or “premium” version with full capabilities. Oftentimes, the “free” or “light” versions contained numerous advertisements for the paid versions. Additional research on these apps was required to determine if the free version of the app was comprehensively usable for the app’s function. The features of both the free and paid versions of the app were evaluated to determine whether the app could be used to its full potential without payment.

Investigating free versus paid versions of the specific apps prompted the development of a secondary, more granular classification system for organizing the apps based on the cost (free or paid), option for a trial or “lite” version, and general app category (eg, health, news, education, and entertainment) gleaned from each app’s website description. When reclassifying apps, predefined Apple categories with similarities were combined to create a new classification, while other broad categories were broken down into more specific reclassification categories. Apple had 24 listed categories on the app store that app developers select from to classify their apps. The classification labeled “Games” was a very broad 25th classification that had many subcategories. However, only 2 games appeared in our search results, which may indicate that games are not prioritized for older adults. In total, 26 categories from Apple were identified, with 11 being reclassified for specificity. Once the data were collected and entered into an Excel spreadsheet, descriptive statistics were calculated.

Privacy policies were only recorded with a “Yes” if they were accessible and available prior to the app being downloaded. As Rosenfeld et al [20] report, the act of downloading a mobile app signifies the disclosure of information, and therefore only apps that offer a privacy policy before downloading should be considered. For each privacy policy, we obtained the specific websites listed for each corresponding app in the App Store and listed these in a password-protected Excel database. If a link to the privacy policy could not be identified through the App Store or the specific app website provided by the App Store, the privacy policy was deemed inaccessible.

## Results

The first 25 links listed within the first 2.5 pages of results to our query of “Apps for Older Adults” comprised our primary data source. Each link contained anywhere from 10 to upwards of 40 apps, totaling 133 different iPhone apps within the first 25 links. Of those applications, 44 (33%) required a fee to operate, with the remaining 89 (67%) usable without any payment (Table 1).

**Table 1:** Presence of characteristics across the mobile apps (N=133).

Characteristic	Yes, n (%)	No, n (%)
Fee charged	44 (33)	89 (67)
Privacy policy	110 (83)	23 (17)
Vetting process	43 (32)	90 (68)
Ranked within domain	69 (52)	64 (48)

**Figure 1:**
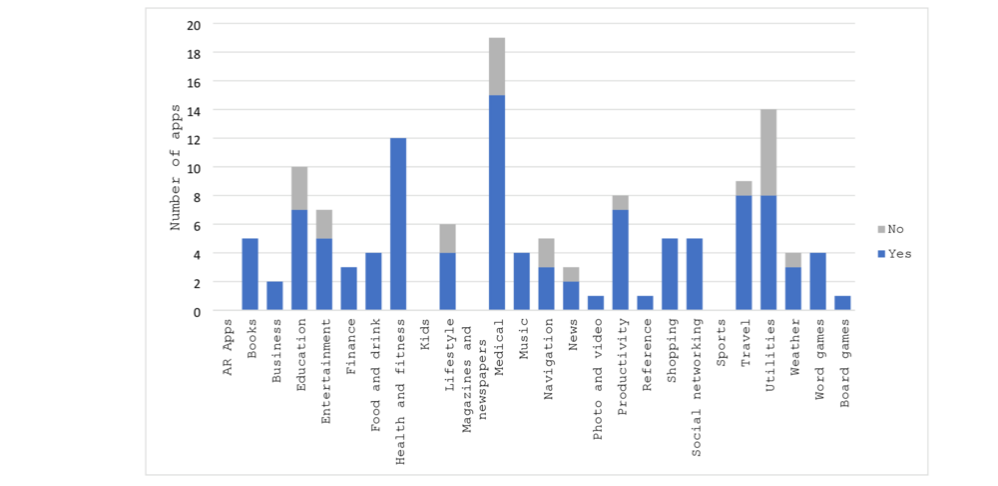
Apps by app store category and existence of a privacy policy.

**Figure 2:**
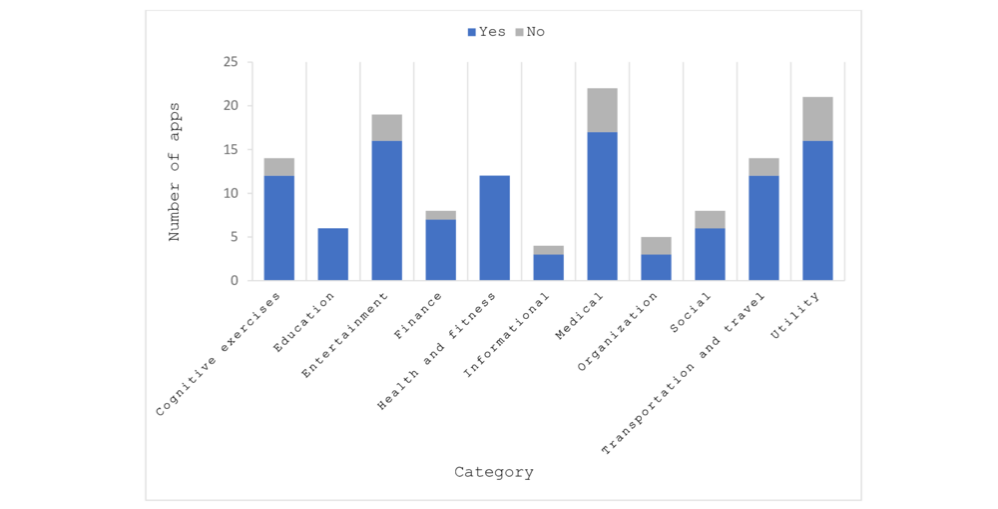
Mobile apps by reclassified category and existence of privacy policy.

Most apps (n=19, 14%) were classified as Medical, which included apps like “Pill Monitor” and “iYogi.” The second most common classification with 14 apps (10%) was *Utilities*, which included apps like “Swiftkey Keyboard” (Figure 1). Figure 1 displays apps within the Apple categories, comparing the number of apps with privacy policies to the total number of apps within a category. After being reclassified, these 2 categories were still the most common with 22 “Health” apps (16%) and 21 apps in the *Utilities* category (16%), respectively (Figure 2). None of the apps were classified as the Apple categories of augmented reality apps, kids, magazines and newspapers, or sports.

In addition, some of the categories predetermined by the Apple Store and selected by the app developers to categorize each app did not align fully with the app’s function or purpose. One example is “Flashlight + Magnifying Glass,” which was categorized as a *Food and Drink* app. Other categories seemed vague and nonspecific to the primary purpose of the apps, such as *Lifestyle*.

Overall, we found that privacy policies were available for the majority of the apps. Out of the total 133 apps analyzed, 110 (83%) had an accessible privacy policy that was available prior to downloading the app, which leaves only 23 apps (17%) with no policy (Table 1). We also found that the *Utilities* category had a lower percentage of apps with an available privacy policy. Within Apple’s categories, only 8 out of 14 (57%) had a privacy policy (Figure 1). Within the reclassified themes, it fared better, where 16 out of 21 apps (76%) had privacy policies (Figure 2). Figure 2 compares the apps with privacy policies to the total number of apps within the reclassified categories.

In both classifications, *Health and Fitness* included 12 apps, with all 12 of them having privacy policies (Figures 1 and 2). The *Medical* category was an area of interest regarding privacy policies as it contained the most apps, many of which dealt with sensitive information, including an individual’s diseases or health history, medications, birthdate, address, and location. Of the 19 apps categorized as *Medical* in the app store, 15 (79%) contained a privacy policy (Figure 1). When reclassified, this percentage remained about the same with 17 out of 22 (77%) including a privacy policy (Figure 2). Despite these high percentages, the fact that they remain below average of all the apps studied could be a cause for concern.

Of the 133 apps analyzed, only 43 (32%) described a vetting process for why the app was recommended (Table 1). Table 1 shows the percentage of the 133 apps for which there was a fee, privacy policy, vetting process, or Apple ranking present. These 43 vetted apps came from only 2 websites that did such. The other 23 links did not offer any explanation for why those apps were recommended. In some cases, it was clear that the recommendations were a marketing tool for a company to get their app promoted. This was apparent in 1 case when SilverSneakers [26] recommended an app called SilverSneakersGo in their “best fitness apps for older adults.” Many of the apps, however, did have recognition in the form of a rating on the app store. An app would show a ranking within its category if it was one of the 200 most popular apps within that category. Of the apps that we analyzed, 69 (52%) were rated (Table 1).

## Discussion

### Overview

Over the past few decades, and even more recently during the COVID-19 pandemic, use of technology by individuals spanning all age groups has increased. This has resulted in an increasingly expansive market for mobile apps of all subtypes. A growing number of older adults are using smartphones and digital applications, with recent reports suggesting that more than half of people aged 65 years and older own a smartphone [7].

Mobile apps are not required to undergo an extensive screening process or security evaluation in order to make it to the market and face even fewer requirements in order to be installed by smartphone users. Mobile apps may be designed with good intentions and may have positive influences on health, lifestyle, and well-being; however, it would be naïve to overlook the potential risks of harm, particularly considering the number of available apps combined with minimal quality control. The misuse of personal information collected on mobile apps is widespread, which can put users at risk [8]. Older adults, who lack familiarity with the web, may be at elevated risk if their personal data are accessible. Older adults are generally concerned about their privacy, but are willing to disclose their information if they are told why it is being collected and what they will receive in return. This trade-off evaluation aligns with the business model of many apps [2,7]. However, this only occurs via a readable, comprehensible privacy policy where the collection, use, and sharing of user data is disclosed.

### Principal Findings

Of the apps that were recommended to older adults from a simple Google search, we found that the majority had a privacy policy. While access to a privacy policy is important, privacy policies are not a safeguard for the end user but, more so a disclosure of how user information will be shared or sold.

The apps were classified into different categories, both by the developers when first launching their apps for public download and reclassified, as appropriate, after having read each app description. The *Medical* category was of greatest interest, considering it required the most sensitive, personal information such as clinical diagnoses and medical history, birth date, and location tracking. In both classification schemes, the majority of the *Medical* apps contained a privacy policy accessible prior to app download; yet, this figure was lower when compared to other classifications. These results are far more optimistic than findings from a review of 300 health apps conducted by Sunyaev et al [27], who found that fewer than a third of health apps in the iTunes and Google Play store had any privacy policy at all in place.

We found that 17 (77%) of the “medical” apps in our data included a privacy policy, which is promising, considering only 91 (30%) included a privacy policy in the 2014 study [27]. The likely cause of this difference lies in our respective samples, but it could be that expectations have shifted over the past few years [28]. The present search only included 19 of the top *Medical* apps, meaning there could be selection bias that skewed the results.

Our results show that a substantial number of apps have an accessible privacy policy, which is a positive outcome in terms of information transparency. Follow-up research is needed to analyze the specific details of the privacy policies to see if they are readable (eg, contain what information is being disclosed), succinct (explicitly explain risks), and can be understood by users.

### Comparison With Prior Work

Similar studies conducted by Das et al [18] and Rosenfeld et al [20] looked at privacy policies for apps targeting youth and older adults with dementia, respectively. Das et al [18] revealed a substantially lower percentage of apps having privacy policies, and of those, the reading level was at a higher grade level (twelfth grade) than that of the average adult, which is around eighth grade. Recommendations from that study included the need for elementary school educational programs to increase awareness of internet safety practices [18]. Rosenfeld et al [20] discovered a far lower percentage of apps included a privacy policy, at 46%, which is concerning for health-related apps that are marketed to potentially vulnerable people.

### Recommendations

The number of people aged 65 years and older is growing rapidly. Most older adults have relatively low technology literacy as well as data literacy, which makes them vulnerable to scams [7,29-31]. Future research could focus on technology and data literacy among older adults. Moreover, technologies that can support the review of privacy policies could be useful in today’s digital age. Improving awareness of the nature and granularity of data being collected by mobile apps could assist older adults to make informed choices when considering whether to use a mobile app. Moreover, providing internet safety and security education as well as developing a policy that serves to protect older adults from potential risks embedded in privacy agreements would be important next steps.

Future evaluations of mobile apps should include a data management assessment, including threats to privacy, such as the elements proposed in Textbox 1. To better understand the possible risks of harm associated with app data collection and management practices, a systematic review process is recommended.

**Privacy aspects to consider in future evaluations of mobile apps.**

Is it clear how data are stored?

Are data shared or sold? If yes, what data?

How is identifiable information protected?

Is bystander data collected?

Is bystander data stored? Shared?

Our study assessed whether a privacy policy existed for each of the mobile apps returned in our search. To determine whether a privacy policy could be a source of harm to the end user, critically evaluating the privacy policy terms is an appropriate next step. Given that medical apps were those which collected the most sensitive data from users, those classified as “medical” should be prioritized. To evaluate the terms described within a privacy policy, one tool to consider is the digital health decision support framework and companion checklist [32]. This framework includes four interconnected domains labeled as (1) Data Management, (2) Privacy, (3) Access and Usability, and (4) Risks and Benefits. The domains are undergirded by principles commonly used in biomedical research ethics including respect for person, beneficence, justice (see Belmont Report), and respect for law and public interest (see Menlo Report) [33,34].

For each of the 19 medical apps, the evaluation might include an assessment to describe the nature and sensitivity of data, identifiability of individual-level data, the purpose of collection of the variable, a description of who would have access to the data (eg, shared with developers, researchers, clinicians, and third parties), and whether there was a statement indicating that a user would give up their rights to file a claim should damages occur. This evaluation of privacy policies could be useful in drawing attention to potential risks as well as making the data-sharing practices more visible to product end users.

### Limitations

Although the first 3 pages in the initial Google search yielded many app results, hundreds of pages were returned as a whole. Additionally, search results are personalized, so different users may have varying results based on individualized returns specific to the digital advertising algorithms of search engines. Our analysis focused only on Apple-specific apps. Including Android apps and predefined categories listed in the Google Play Store may provide additional categorizations. In addition, the Apple apps we investigated did not have a vetting process as to why they were recommended. Future research could focus on these websites to see if there is a related conflict of interest (eg, financial incentives) that explains why these certain websites advertise links while not providing any evidence for the recommendation.

### Conclusions

The market for mobile apps has dramatically increased concurrently with the rapid growth of people older than 65 years of age [5]. Older adults may have limited technology literacy and require assistance to use their smartphones, which makes them vulnerable to exploitation by app developers and app manufacturers [7]. Our findings show that the majority of apps that are recommended for seniors do, in fact, include access to a privacy policy. To what extent those policies are read or understood by older adults is not known. Evaluating privacy policy content and potential risks of harm to end users, particularly harms linked to medical app data management practices, is an important next step.
